# Prevalence and contributing factors of anaemia among children aged 6–24 months and 25–59 months in Mali

**DOI:** 10.1017/jns.2023.93

**Published:** 2023-11-09

**Authors:** Tafere Gebreegziabher, Saran Sidibe

**Affiliations:** Food Science and Nutrition, Department of Health Sciences, Central Washington University, 400 E University Way, Ellensburg, WA 98926-7571, USA

**Keywords:** Anaemia, Contributing factors, Children, Mali

## Abstract

Although considerable global initiatives have been undertaken to tackle anaemia, its prevalence continues to be high in sub-Saharan African nations. In Mali specifically, anaemia represents a significant and pressing public health issue. The purpose of the present study was to examine the key risk factors related to anaemia among children aged 6–24 months (younger age group) and 25–59 months (older age group). We used the Mali 2018 Demographic and Health Survey data, collected from 8861 mothers with children under five. Logistic regression was used to assess the risk factors for childhood anaemia. The results suggest that the prevalence of anaemia was 88 % in the younger and 76 % in the older age groups. The risk factors unique to the younger age group were malaria (OR 4⋅05; CI 0⋅95, 11⋅3) and place of residence (OR 0⋅55; CI 0⋅32, 0⋅94), while for the older age group, they were morbidity (OR 1⋅91; CI 1⋅12, 3⋅24), drinking from a bottle (OR 1⋅52; CI 1⋅04, 2⋅22), and micronutrient intake (OR 0⋅61; CI 0⋅40, 0⋅91). Risk factors that significantly contributed to both age groups include breastfeeding, deworming, maternal anaemia, maternal education, and wealth index. Anaemia also varied by region. The widespread prevalence of anaemia can be attributed to a multitude of factors. In addressing this issue, it is imperative to acknowledge the unique characteristics of specific regions and rural areas, where the incidence of anaemia surpasses the national average. Therefore, any intervention efforts should be tailored to the specific needs and challenges of these areas.

## Introduction

Anaemia is a medical condition characterized by a deficiency in the number of red blood cells or a decrease in the amount of haemoglobin per unit volume of blood circulating in the body's peripheral vessels^(1)^. Anaemia is a significant worldwide public health issue that predominantly impacts specific vulnerable groups. According to the World Health Organization (WHO), currently, approximately 40 % of children aged 6–59 months are affected by anaemia on a global scale, and approximately 269 million children are experiencing significant health challenges as a result of anaemia^(2)^. In 2019, over 60 % of children aged 6–59 months in Africa experienced anaemia and more than 40 % were in the category of severe anaemia^(2,3)^. This condition exposes them to various serious consequences, such as a heightened vulnerability to infections and mortality, impaired cognitive abilities, excessive tiredness, hindered growth and development, and reduced earning potential later in life^(2)^.

Anaemia in children aged 6–59 months, as defined by the WHO, is characterized by a haemoglobin concentration below 110  g/l^(4)^. It is essential to recognize that while iron deficiency is a significant factor in this global health issue, it is not the sole cause. Approximately half of all anaemia cases worldwide are linked to iron deficiency, while other contributing factors include deficiencies in vitamin B12, folate, or vitamin A, genetic disorders, parasitic infections, and chronic inflammation. These factors can be further intensified by low socio-economic status, inadequate education, poor sanitation, and hygiene conditions^(2,5)^.

In a study encompassing demographic and health survey data for thirty-nine African countries and focusing on children under the age of five, it was reported that the socio-economic status (wealth and education) of mothers has an impact on childhood anaemia. Children from economically disadvantaged households and those whose mothers lacked formal education or had only completed primary education had a higher occurrence of anaemia compared to children from more affluent households and those whose mothers had completed secondary education or higher^(6)^. Mali is one of the countries where children under the age of five are highly affected by anaemia. In 2022, Mali had one of the highest rates, with 82 % of children under the age of five being affected by anaemia^(6)^. Other contributing factors to anaemia among Malian children include malaria, poor sanitation, parasitic infestations, and infections^(7,8)^. As a result, the immune system is compromised, potentially raising vulnerability to morbidity and mortality^(9)^.

Although young children under the age of five are susceptible to illness and mortality, the first 2 years of life are considered the most critical period for growth and development. For instance, infants primarily rely on breast milk to fulfil their daily nutritional requirements, while older children incorporate complementary foods into their diet. Moreover, factors such as exposure to diverse foods, feeding and childcare practices, hygiene and sanitation, and social interaction significantly impact the well-being of children^(10)^. Consequently, in this study, our objective was to examine the prevalence and factors associated with anaemia by dividing children under the age of five into two age groups: 6–24 months and 25–59 months. To the best of our knowledge, this is the first study conducted in Mali that specifically analyses the risk factors for anaemia in these two age groups using the 2018 DHS data.

## Data source and methodology

### Data source

The current study employed secondary data from the Malian Demographic and Health Survey (MDHS 2018). The MDHS is a comprehensive cross-sectional survey conducted through a multistage complex sampling approach^(11)^.

### Study design and study population

The sampling design ensures accurate estimations at the national level, as well as for urban and rural areas, including each of the eight regions and the capital city of Mali, Bamako. However, due to challenges in reaching rural populations and security concerns, only the urban area of the Kidal region was included in the survey^(11)^. The current study specifically concentrated on the data referred to as the ‘kid's file,’ which encompassed a comprehensive collection of health and socio-demographic data concerning mothers and their children under the age of five. For the present analysis, a total sample size of 8861 children aged 6–59 months was considered. Infants below the age of 6 months were excluded from the analysis as the MDHS did not measure haemoglobin levels for this particular age group. The detailed description of methods, design, instruments, participants, and sampling frame was previously published by the MDHS program at: https://dhsprogram.com/methodology/survey/survey-display-517.cfm

The MDHS adhered to globally recognized standard protocols, data collection tools, and procedures. Participation in the survey was voluntary, and the microdata collected were made accessible to the public by DHS.

### Outcome and exposure variables

The outcome variable anaemia was defined by haemoglobin (Hgb) level < 110 g/l^(4)^. The haemoglobin was measured using a HemoCue®201 analyzer which was adjusted for altitude^(11)^. Anaemia level was further defined into non-anaemia (Hgb level ≥ 110 g/l); mild anaemia (Hgb level 100–109 g/l); moderate anaemia (Hgb level 70–99 g/l); and severe anaemia (Hgb level < 70 g/l).

Exposure variables were divided into child characteristics (sex of child, morbidity, breast feeding, if child drank from bottle the night preceding the survey, deworming, malaria, micronutrient intake, and immunization); maternal characteristics (maternal anaemia level (Hgb < 120 g/l), maternal education, maternal age, maternal age at first birth, and maternal work status); household factors (household size, wealth index, and source of drinking water). The community-level variables were place of residence and region. The assessment of morbidity condition relied on self-reported indications and symptoms of diarrhoea and acute respiratory infection. These symptoms encompassed coughing, fever, and chest congestion experienced within a 2-week period prior to the survey date. For deworming the information was collected if the child was given drugs for intestinal parasites. Similarly, for micronutrient intake, the information was collected if the child was given micronutrient supplement in the last 6 months prior to data collection. Malaria was assessed based on self-reported indications and whether the child had received one or more of the drugs used for malaria treatment. The estimation of household income was done through a self-reported assessment of the household's wealth index based on assets. All remaining variables were used in their original coded format by MDHS. The independent variables were selected based on review of literature and model-fitting criteria.

### Statistical analysis

Data analysis was conducted using IBM SPSS Statistics version 23, a statistical software package. All analyses were weighted to account for sampling probabilities. Descriptive analysis was performed to examine the characteristics of the study sample. To investigate the relationships between the explanatory variables and the outcome variable, multivariate logistic regression analyses were employed. For each of the two age groups (6–24 months and 25–59 months), a regression model was constructed using stepwise selection. The selection of explanatory variables for the regression analysis was performed based on previous research findings and model-fitting procedures. To assess multicollinearity among the explanatory variables, we utilized the variance inflation factor, and variables with a variance inflation factor exceeding 2⋅5 were eliminated from the analysis. The model fit was evaluated using the Hosmer–Lemeshow test. Odds ratios (OR) and 95 % confidence intervals (CI) were calculated to determine the likelihood of being anaemic associated with each factor in the logistic regression model. Statistical significance was considered if the *P*-value was less than 0⋅05.

## Results

### Characteristics of study participants

**Fig. 1.**
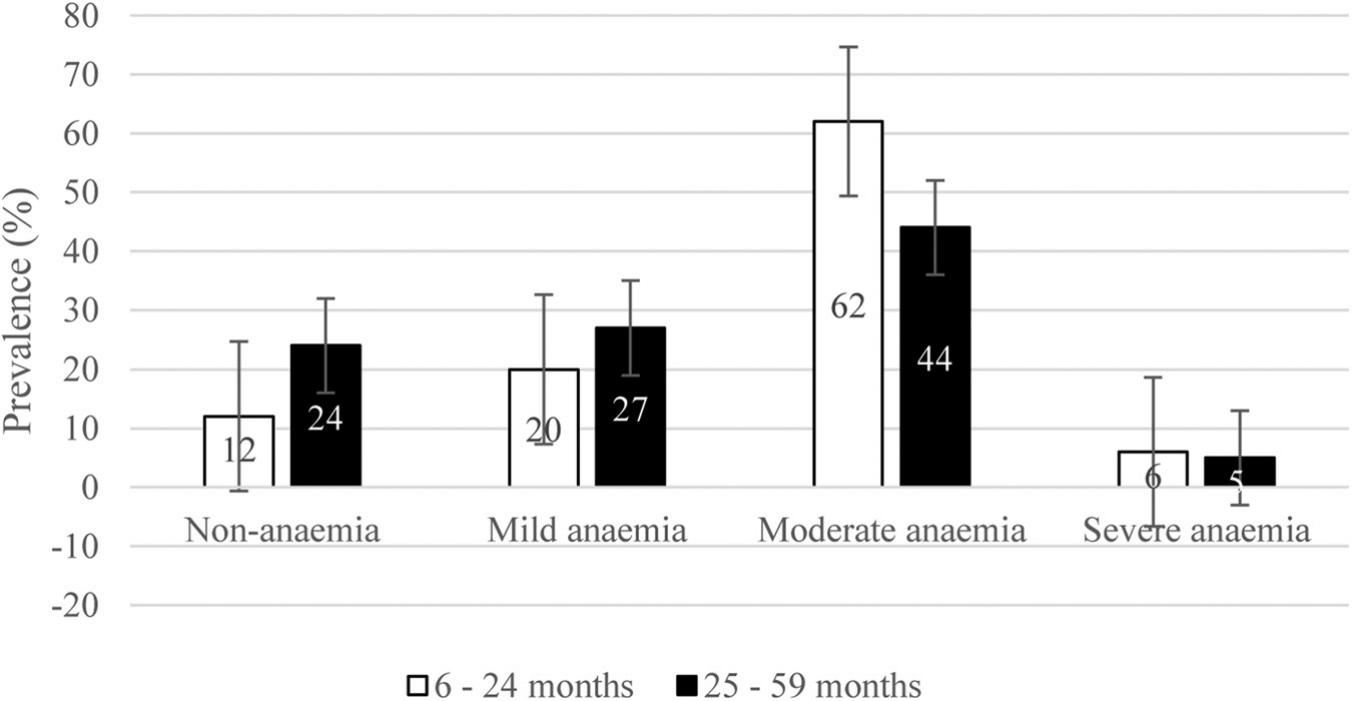
Prevalence of anaemia among children aged 6–24 months and 25–59 months (*n* 8861).

As presented in Table 1, most of the study participants were rural inhabitants. More than 17 % of the younger age group and more than 14 % of the older age group were small size at birth. Prevalence of anaemia was high in both age groups. Most of the children in both age groups have experienced varying levels of anaemia (Fig. 1). Almost 40 and 27 % of the younger and older groups, respectively, exhibited symptoms of morbidity indicators. Approximately 35 % of the younger age group and 42 % of the older age group received intestinal parasite treatment within 6 months before the survey. The prevalence of malaria was 14⋅3 and 18⋅6 % in the younger and older age group, respectively. In both groups, most households lack access to a piped water source. Additionally, most of the mothers in both groups fell within the age category of 25–34 years old. More than 70 % of the mothers in the younger age group and 75 % of the mothers in the older age group had no education. Based on the wealth index category, almost 38 % of the younger age group and over 41 % of the older age group were classified as ‘poor,’ while a similar proportion of women were categorized as ‘rich’. Prevalence of anaemia was high in both groups.

**Table 1.** Socio-economic and demographic characteristics of households by children's age group (*n* 8861), MDHS, 2018

Variable	6–24 months (*n* 3217)		25–59 months (*E* 5644)	
	*n*	%	*n*	%
Place of residence				
Urban	833	25⋅9	1410	25
Rural	2384	74⋅1	4234	75
Child sex				
Male	1653	51⋅4	2856	50⋅5
Female	1564	48⋅6	2788	59⋅4
Size of child at birth				
Small	524	17⋅2	765	14⋅3
Average	1519	49⋅8	2772	52⋅0
Large	1009	33⋅0	1798	33⋅7
Source of water				
Not piped	2121	65⋅9	3843	68⋅1
Piped	1096	34⋅1	18⋅1	31⋅9
Malaria^a^				
No	588	85⋅7	698	81⋅4
Yes	98	14⋅3	159	18⋅6
Child morbidity^b^				
No	1850	60⋅5	3720	73⋅3
Yes	1206	39⋅5	1354	26⋅7
Deworming^c^				
No	1952	65⋅3	2885	58⋅1
Yes	1037	34⋅7	2082	41⋅9
Immunization				
No	533	17⋅6	310	20⋅6
Yes	2496	82⋅4	1198	79⋅4
Maternal anaemia				
Not anaemic	598	38⋅1	999	35⋅4
Anaemic	973	61⋅9	1823	64⋅6
Wealth index				
Poor	1221	37⋅9	2355	41⋅7
Middle	717	22⋅3	1232	21⋅8
Rich	1279	39⋅8	2057	36⋅5
Maternal age				
15–24	1122	34⋅9	1411	25⋅0
25–34	1455	45⋅2	2826	50⋅1
35–49	640	19⋅9	1407	24⋅9
Maternal education				
No education	2271	70⋅6	4231	75⋅0
Primary	407	12⋅7	670	11⋅9
Secondary	493	15⋅3	684	12⋅1
Higher	46	1⋅4	59	1⋅0

a Received one or more drugs for malaria symptoms.

b Child had one or more of the listed health problems in the last 2 weeks (fever, cough, short rapid breath).

c Drugs for intestinal parasite in last 6 months.

### Correlates of childhood anaemia

Table 2 reveals associations with childhood anaemia, including child morbidity, feeding practices, malaria, parasite medication, micronutrients, maternal factors, wealth, education, residence, and region. While both age groups had similar variables, their significance differed. Importantly, gender was not associated with anaemia in either group.

**Table 2.** Logistic regression for selected predictors of anaemia among under five children, MDHS, 2018

	Children 6–24 months (*n* 3217)		Children 25–59 months (*n* 5644)	
	OR (95 % CI)	*P*-value	OR (95 % CI)	*P*-value
Child sex				
Male	1		1	
Female	0⋅91 (0⋅66, 1⋅25)	0⋅55	0⋅87 (0⋅72, 1⋅05)	0⋅14
Child morbidity^a^				
No	1		1	
Yes	1⋅00 (0⋅69, 1⋅43)	0⋅99	1⋅91 (1⋅12, 3⋅24)	**0⋅017**
Drank from bottle^b^				
No	1		1	
Yes	0⋅77 (0⋅43, 1⋅38)	0⋅381	1⋅52 (1⋅04, 2⋅22)	**0**⋅**024**
Breast feeding				
No	1		1	
Yes	1⋅70 (1⋅17, 2⋅35)	**0**⋅**0004**	1⋅75 (1⋅02, 3⋅01)	**0**⋅**044**
Malaria^c^				
No	1		1	
Yes	4⋅05 (0⋅95, 11⋅3)	**0**⋅**031**	1⋅67 (0⋅86, 3⋅25)	0⋅13
Deworming^d^				
No	1		1	
Yes	1⋅75 (1⋅06, 2⋅98)	**0**⋅**02**	1⋅62 (1⋅17, 2⋅12)	**<0**⋅**001**
Micronutrient intake^e^				
No	1		1	
Yes	0⋅55 (0⋅25, 1⋅23)	0⋅144	0⋅61 (0⋅40, 0⋅91)	**0**⋅**032**
Maternal anaemia				
Not anaemic	1		1	
Anaemic	1⋅73 (1⋅22, 2⋅45)	**0**⋅**002**	2⋅01 (1⋅30, 3⋅06)	**0**⋅**002**
Wealth index				
Poor	1		1	
Middle	1⋅18 (0⋅70, 1⋅99)	0⋅54	0⋅68 (0⋅37, 1⋅25)	0⋅212
Rich	0⋅64 (0⋅43, 0⋅90)	**0**⋅**031**	0⋅67 (0⋅40, 1⋅11)	**0**⋅**122**
Maternal education				
Illiterate	1		1	
Literate	0⋅73 (0⋅52, 0⋅95)	**0**⋅**045**	0⋅76 (0⋅62, 0⋅94)	**0**⋅**011**
Place of residence				
Rural	1		1	
Urban	0⋅55 (0⋅32, 0⋅94)	**0**⋅**021**	0⋅79 (0⋅56, 1⋅12)	0⋅187
Region				
Bamako	1		1	
Kayes	0⋅81 (0⋅37, 1⋅75)	0⋅59	2⋅001 (1⋅22, 3⋅28)	**0**⋅**006**
Koulikoro	0⋅82 (0⋅37, 1⋅82)	0⋅63	1⋅85 (1⋅13, 3⋅01)	**0**⋅**014**
Sikasso	1⋅45 (0⋅68, 3⋅08)	0⋅34	1⋅69 (1⋅06, 2⋅69)	**0**⋅**027**
Segou	0⋅99 (0⋅44, 2⋅25)	0⋅90	1⋅95 (1⋅19, 3⋅20)	**0**⋅**008**
Mopti	0⋅91 (0⋅39, 2⋅12)	0⋅83	1⋅24 (0⋅75, 2⋅06)	0⋅403
Toumbouctou	0⋅55 (0⋅26, 1⋅19)	0⋅13	0⋅76 (0⋅47, 1⋅22)	0⋅25
Gao	0⋅32 (0⋅15, 0⋅65)	**0**⋅**002**	0⋅60 (0⋅38, 0⋅96)	**0**⋅**033**
Kidal	0⋅40 (0⋅19, 0⋅80)	**0**⋅**01**	1⋅09 (0⋅72, 1⋅66)	0⋅66

OR, odds ratio. Bold values indicate significant, and the exact P-values are given.

a Child had one or more of the listed health problems in the last two weeks (fever, cough, short rapid breath).

b Received any liquid (including breast milk) or semi-solid food from a bottle with nipple/teat.

c Received one or more drugs for malaria symptoms.

d Drugs for intestinal parasite in last 6 months.

e Child has taken one or more of the listed micronutrients (iron, zinc, or vitamin A).

In the older age group, child morbidity demonstrated a significant association with anaemia (OR 1⋅91; 95 % CI 1⋅12, 3⋅24), whereas this association was not observed in the younger age group. Similarly, drinking from a bottle the night before the survey was correlated with anaemia in the older age group (OR 1⋅52; 95 % CI 1⋅04, 2⋅22). Furthermore, the odds of experiencing anaemia were 4⋅05 times higher in the younger age group who had taken one or more types of medicine for malaria treatment (95 % CI 0⋅95, 11⋅3).

In both groups, children receiving parasite treatment had higher odds of anaemia. In the older group, those taking micronutrient supplements during the reference period had a 39 % lower anaemia risk (OR 0⋅61; 95 % CI 0⋅40, 0⋅91). Maternal anaemia was linked to childhood anaemia in both groups, with 1⋅73 (95 % CI 1⋅22, 2⋅45) higher odds in the younger group and 2⋅01 (95 % CI 1⋅30, 3⋅06) times higher odds in the older group. High-income households and maternal literacy were also associated with reduced anaemia risk in both age groups.

Both residence and region predicted anaemia. Among urban children, both age groups had lower odds of anaemia, though this was not significant in the older group. In contrast to Bamako, the capital city, children in Kayes, Koulikoro, Sikasso, and Segou regions had higher odds of anaemia in the older group. However, children in Gao and Kidal regions had lower odds of anaemia in the younger group. Additionally, the older group showed lower anaemia odds for children in the Gao region.

## Discussion

Anaemia in children under 5 years has been high for decades in Mali. Because of various efforts to prevent and treat infections such as malaria, which is one of the contributing factors to anaemia in Mali, the percentage of children suffering from severe anaemia has decreased for over a decade. In 2010, the prevalence of severe anaemia was 26⋅3 %. This number decreased to 20⋅6 % in 2012 and continued to decline to 19⋅9 % in 2015^(12)^. In the current analysis, the percentage of children under 5 years experiencing severe anaemia was as low as 5⋅5 %. However, the overall prevalence of anaemia does not show any pattern of decline. In a survey conducted between 2010 and 2018 in fifteen African countries, nearly 65 % of children in sub-Saharan Africa (SSA) were anaemic, with the highest percentage observed in Mali at 82 %^(13)^. Similarly, in the current analysis, the overall prevalence of anaemia in children under five was 82 %, with 88 % in the younger age group.

Mali, a low-income country in SSA, has a predominantly rural population, with over 55 % residing in rural areas^(14)^. Similar to many African nations, rural Mali faces challenges such as limited access to education, economic resources, and healthcare facilities^(15)^. In our current analysis, more than 74 % of the population lives in rural areas, nearly 73 % have no formal education, and almost 40 % are classified as poor based on the wealth index. Our study reveals that various factors at the child, maternal, household, and community levels contribute to the high prevalence of childhood anaemia in Mali. The prevalence of anaemia varies significantly by region and residence area, with specific regions and rural areas showing a strong association with an increased risk of childhood anaemia. These associations may be attributed to disparities in resource access, education levels, cultural and dietary practices, as well as income disparities.

Compared to children whose mothers were poor or illiterate, children with wealthy or educated mothers have a reduced risk of experiencing anaemia. Furthermore, maternal anaemia was found to be negatively correlated with childhood anaemia, which supports previous studies^(10,16,17)^. From a global perspective, regions with higher levels of socio-economic development show a lower prevalence of anaemia^(18)^. This underscores the importance of socio-economic status in determining childhood anaemia and highlights the need to address economic inequalities as a crucial factor in effectively combating anaemia.

The prevalence of anaemia differed between the two age groups, and so did the factors associate with it, although they had some similarities. In the older age group, children who had fallen ill within the 2 weeks before the survey had a higher likelihood of anaemia. This contrasts with findings in Ethiopia, where a higher likelihood of anaemia was observed in the younger age group^(10)^. The illnesses reported included coughing, fever, and chest congestion. These results align with a study in rural Indonesia that confirmed a link between current or recent episodes of diarrhoea and anaemia in children under the age of five, even after controlling for other factors contributing to both diarrhoea and anaemia^(19)^.

Poor sanitation and hygiene-related diarrhoeal diseases are prevalent in Mali. There is a consistent link between lower quality and limited access to drinking water sources and the occurrence of waterborne diseases^(20)^. Additionally, infant feeding practices have been identified as a cause of diarrhoea and diarrhoeal mortality in SSA in general, and specifically in Mali^(21)^. In our analysis, children who drank from a bottle the night before the survey had higher odds of experiencing anaemia in the older age groups. Similarly, in Mexico, prolonged bottle feeding was associated with iron deficiency anaemia^(22)^. Furthermore, various studies have highlighted the significant role of child-feeding practices in contributing to nutrient inadequacy, ultimately resulting in iron deficiency anaemia. In regions such as Mali and other West African countries, there is growing concern about the impact of introducing commercial snack foods and beverages as complementary foods for children. These products tend to be high in calories but lacking in essential nutrients, which can adversely affect child nutritional outcomes^(23)^. Similarly, in Brazil, the introduction of complementary feeding for young children has been linked to cases of iron deficiency anaemia, primarily attributed to a lack of dietary diversity^(24)^.

Despite the acknowledged benefits of breastfeeding (BF) in meeting the nutritional needs of young children and promoting overall health, there have been inconsistent findings regarding its role in reducing the burden of anaemia. For instance, a retrospective cohort study conducted in Japan found a high prevalence of anaemia among infants who were exclusively breastfed^(25)^. In Bangladesh, exclusive BF for 6 months was associated with low levels of zinc and iron, as well as a higher prevalence of anaemia^(26)^. Similarly, in Mexico, BF at 9 months was linked to increased odds of anaemia, particularly among children whose mothers had a history of anaemia^(27)^. Our findings align with these reports, further supporting the notion that the relationship between BF and anaemia is complex and may be context dependent.

In the present analysis, the consumption of micronutrients has been associated with lower odds of anaemia among older age groups. In regions where anaemia prevalence is high, the WHO recommends the fortification of home-cooked foods with micronutrient powders (MNP) to enhance the iron status of young children and reduce anaemia^(28)^. While information specifically regarding the practice of home fortification with MNP in Mali is limited, a systematic review of MNP trials in low-income countries indicated an overall reduction in anaemia rates^(29)^. One study reported that over 80 % of parents reported having given MNP to their child, with 65 % having administered MNP for 4 or more days in the week prior to data collection.^(30)^.

In Mali, young children were affected by various infectious diseases such as meningitis and malaria. Due to the significant incidence of meningitis, Mali is classified as one of the African countries within the region known as the ‘meningitis belt’^(31)^.

Despite progress toward eliminating malaria in the past decade, malaria remains a public health problem in SSA in general, and specifically in Mali, to the extent that additional interventions are necessary^(32,33)^. In the present analysis, over 16 % of children under five received one or more types of drugs to treat malaria symptoms. Moreover, the likelihood of experiencing anaemia was higher among children who received any of those drugs in the younger age groups. Similarly, malaria has been identified as one of the main contributing factors to childhood anaemia in tropical regions^(34)^, which was also evident in Cameroon^(35)^. Regardless of whether it occurs in settings with higher or lower transmission rates, young children are the most vulnerable groups to suffer from anaemia caused by malaria infection^(36)^. The pathogenesis of malarial anaemia is multidimensional. Malaria, being an intraerythrocytic parasite, leads to obligatory destruction of red blood cells. However, a more significant factor is the accelerated destruction of non-parasitized red blood cells, which occurs parallel to the severity of the disease^(37)^. It is estimated that approximately 90 % of the acute anaemia resulting from a single infection is attributed to the loss of unparasitized red blood cells^(38)^. In severe falciparum malaria, a substantial parasite burden leads to the rapid development of anaemia^(36)^.

Deworming is an effective intervention for improving children's nutritional status and health. The available evidence suggests that the deworming program implemented for school children in Ethiopia has yielded promising results in reducing parasitic infestation and the prevalence of anaemia^(39,40)^. Additionally, an analysis of data from the Demographic and Health Surveys revealed that Ethiopian children who were not treated for intestinal parasites were more likely to experience anaemia^(10)^. Moreover, administering a deworming treatment with iron supplementation during pregnancy has been shown to reduce anaemia in infants^(41)^.

However, our findings differ from these results. In a randomized controlled trial involving Angolan children, treating intestinal parasites with albendazole did not demonstrate a significant difference compared to the control group, but it did show a decreased likelihood of developing anaemia over time^(42)^. A meta-analysis examining prenatal deworming also did not find a beneficial effect on childhood anaemia^(43)^. This can be attributed to the fact that anaemia has multiple causes, and if other factors contributing to anaemia, such as inadequate supplementation or malaria, are not addressed, the benefits of parasite control may be diminished^(44)^. Furthermore, it is important to consider that deworming can reveal the presence of infections in children, and therefore, the timing of intervention in relation to the severity of anaemia can have an impact.

### Strengths and limitations

These findings were based on a large sample size that covered all regions in Mali, allowing for generalization to the larger population. The analysis examined a wide range of variables, including factors related to children, mothers, and socio-economic status, which directly or indirectly impact childhood anaemia. This information can be valuable for planning, targeting, and monitoring childhood anaemia at a national level, particularly given the limited availability of studies on this subject. However, the present study has several noteworthy limitations. Firstly, the utilization of a cross-sectional design impedes the establishment of a causal relationship between various factors and childhood anaemia. Secondly, the high prevalence of anaemia within the study population, approximately 82 %, diminished the variability in certain expected variables, thereby challenging the observation of significant associations with the outcome measures. Furthermore, reliance on self-reported data introduces the potential for recall and misclassification biases, further underscoring the need for cautious interpretation of the findings.

## Conclusion and policy implication

In summary, our findings highlight the alarming prevalence of anaemia among children under the age of five, with rates as high as 88 % in the younger age group and 76 % in the older age group. Anaemia is a complex condition with multiple contributing factors, making it difficult to pinpoint a single cause. Nonetheless, our analysis revealed that factors linked to diarrhoeal diseases, including their causes and related elements, significantly influence the likelihood of childhood anaemia.

To combat this issue, it is crucial to focus on strengthening both nutrition-sensitive interventions, such as health education and promoting maternal knowledge regarding WASH (water, sanitation, and hygiene), as well as nutrition-specific interventions like providing micronutrient supplementation, deworming treatments, and encouraging healthy feeding practices. These comprehensive measures have the potential to effectively reduce high anaemia prevalence in the country. Special emphasis should be placed on rural households and disadvantaged regions with rates surpassing the national average. Hence, improving healthcare access, empowering women, and alleviating poverty in these marginalized areas is paramount importance. Our study also underscored the significance of region and place of residence as predictors of anaemia, highlighting the necessity for tailored interventions that consider the unique characteristics of each specific area.
